# Beneficial Metabolic Effects of Praliciguat, a Soluble Guanylate Cyclase Stimulator, in a Mouse Diet-Induced Obesity Model

**DOI:** 10.3389/fphar.2022.852080

**Published:** 2022-03-04

**Authors:** Chad D. Schwartzkopf, John R. Hadcock, Guang Liu, Peter Germano, Julien Roux, Courtney M. Shea, Emmanuel S. Buys, Juli E. Jones

**Affiliations:** ^1^ Cyclerion Therapeutics, Cambridge, MA, United States; ^2^ Biomeostasis, Marseille, France

**Keywords:** praliciguat, cGMP (cyclic GMP), nitric oxide, metabolic disease, thermoneutrality, obesity, soluble guanylate cyclase (sGC)

## Abstract

Praliciguat is a soluble guanylate cyclase stimulator that elicits hemodynamic, anti-inflammatory, and antifibrotic effects in preclinical models of metabolic dysfunction. We assessed the metabolic effects of praliciguat in a mouse diet-induced obesity (DIO) model housed at thermoneutrality. At 6 weeks old, male C57BL/6N mice were either maintained on low-fat diet (LFD, lean mice) or placed on 60% high-fat diet (HFD, DIO mice). At 14 weeks old, the DIO mice were either maintained on HFD or switched to HFD with praliciguat (6-mg/kg). Day 28 samples were collected for biomarker analysis. In a second study under the same paradigm, indirect calorimetry was performed on days 8, 9, 20, 21, 32, and 33 and an oral lipid tolerance test (LTT) on day 38. Mice treated 28 days with praliciguat had lower levels of fasting plasma insulin, C-peptide, triglycerides, and HOMA-IR (homeostatic model assessment for insulin resistance) than DIO controls. In addition, energy expenditure was higher in praliciguat-treated than in DIO control mice on days 9, 20, 32, and 33; and day-38 triglycerides were lower. HFD-induced increases in gene expression of liver TNF-ɑ, lipoprotein lipase (*Lpl*), and patatin-like phospholipase domain-containing protein 3 (*Pnpla3*) in control DIO mice were attenuated in praliciguat-treated DIO mice. The positive metabolic effects observed in praliciguat-treated mice were associated with the restoration of liver PI3K (pAKT-Thr308) signaling, but not MAPK (pERK). In conclusion, praliciguat-treated DIO mice had increased energy utilization, improved insulin sensitivity, and lower plasma triglycerides. These results illustrate metabolic effects associated with praliciguat treatment in DIO mice.

## Introduction

Obesity is an increasingly urgent global health issue. Between 1975 and 2016, the obesity rate nearly tripled, and the World Health Organization estimated that 39% of adults are overweight and 13% are obese. Thus, there are now more adults who are overweight in the world than are underweight (NCD Risk Factor Collaboration (NCD-RisC), 2016). Obesity—defined by a body mass index (BMI) > 30 kg/m^2^—is the second leading cause of preventable death in the United States (US); it decreases life expectancy by up to 10 years, and accounts for 20% of US total healthcare costs (Afshin et al., 2017). Obesity is a major risk factor for heart disease, stroke, diabetes, non-alcoholic steatohepatitis, musculoskeletal disorders, and adverse outcomes of COVID-19. Some cancers and 90% of type II diabetes cases are accompanied by increased BMI. Therapies for obesity do not reliably produce meaningful weight loss or weight maintenance. For instance, diet and exercise rarely result in durable weight loss due to poor compliance. Bariatric surgery is effective for weight loss but carries a risk of major complications (Aminian et al., 2018; Srivastava and Apovian, 2018). Currently, the few approved drug therapies promote mild weight loss (Srivastava and Apovian, 2018) while one therapy (semaglutide) elicits moderate weight loss (Eliaschewitz and Canani, 2021; Wilding et al., 2021).

In recognition of the expanding views on metabolic health, the term “metabolic syndrome” has been recently adopted to broadly define metabolic dysfunction (Han and Lean, 2016) and refers to a cluster of conditions that increase the risk of cardiovascular disease, diabetes, dyslipidemia, and stroke (Saklayen, 2018). Hallmarks of metabolic syndrome, including insulin resistance and elevated triglycerides, can be independent of body weight. Although BMI is an important aspect of metabolic health, increasing evidence indicates body fat distribution is a more significant marker of overall health. For instance, the waist-to-hip ratio—a surrogate for visceral adipose-to-subcutaneous-adipose tissue ratio—is associated with insulin resistance even in “normal weight” individuals and is highly predictive of cardiovascular disease (Murray, 2006; Benites-Zapata et al., 2019). In fact, individuals with higher visceral fat levels are more metabolically unhealthy and have worse health outcomes versus individuals with the same BMI but whose adipose tissue deposition is largely subcutaneous (Kragelund and Omland, 2005; Muller et al., 2012; Thomas et al., 2012; Aminian et al., 2018; Srivastava and Apovian, 2018). Thus, identifying effective therapies that increase metabolic health do not necessarily need to induce weight loss; rather, increasing insulin sensitivity is critical (Lebovitz, 2001).

The nitric oxide (NO)–soluble guanylate cyclase (sGC)–3′,5′-cyclic guanosine monophosphate (cGMP) signaling pathway is increasingly recognized as a potential therapeutic target for human diseases with a metabolic component (Buys et al., 2018). Praliciguat (IW-1973, PRL) is a small-molecule sGC stimulator that potentiates the NO-cGMP pathway (Banijamali et al., 2020; Hanrahan et al., 2020a; Liu et al., 2020; Shea et al., 2020); thereby increasing cGMP production leading to activation of cGMP-dependent protein kinase to modulate physiological mechanisms such as vasodilation, fibrosis, and inflammation (Buys et al., 2018). In animal studies, praliciguat treatment resulted in increased cGMP levels in many tissues (Tobin et al., 2018). In preclinical models of cardiorenal disease, praliciguat treatment preserved cardiac function, attenuated cardiac hypertrophy and kidney damage, and lowered levels of inflammation and fibrosis biomarkers (Tobin et al., 2018; Hall et al., 2019; Shea et al., 2020). Further, in an exploratory phase 2 study in 26 patients with type 2 diabetes and hypertension on standard-of-care therapy, 14 days of oral praliciguat showed positive trends on blood pressure, plasma glucose and cholesterol levels (Hanrahan et al., 2020b). In another phase 2 study, in patients with diabetic kidney disease on standard-of-care agents for blood pressure and metabolic control, 12 weeks of once-daily praliciguat reduced HbA1c, cholesterol and blood pressure (Hanrahan et al., 2020a).

Diet-induced obesity (DIO) models are widely used in obesity, diabetes, and metabolic disease research (Vickers et al., 2011; Guo, 2014). In DIO mouse model studies with praliciguat, C57BL/6N male mice were fed a high-fat diet (HFD; 60% kilocalories from fat) starting at 6 weeks of age. Animals exposed to this diet develop metabolic syndrome within 3 weeks that includes increased adiposity, fatty livers (NAFLD), insulin resistance, and leptin resistance. With sustained exposure to this diet, adiposity and insulin resistance continue to increase in these mice yet rarely progress to severe insulin resistance and diabetes (Avtanski et al., 2019).

In all mammalian species, thermal stress alters basal metabolic rate (Gordon, 2012). For laboratory experiments in rodents, the standard vivarium temperature (∼22°C) is below a rodent’s thermoneutral zone and therefore introduces thermal stress that can lead to the activation of brown adipose tissue (BAT), which increases brown fat thermogenesis and fatty acid oxidation (Hoffmann et al., 2015). Nonclinical experimental conditions and/or agents that activate BAT in a rodent can elicit results that may not translate to humans, since humans are not typically subjected to thermal stress, nor do they readily increase BAT thermogenesis (Marlatt and Ravussin, 2017). In standard animal housing conditions, administration of an sGC stimulator increases BAT activation in mice (Hoffmann et al., 2015). To explore the effects and mode of action of praliciguat on metabolism, and to mitigate any involvement of BAT activation, we administered praliciguat to DIO mice housed under thermoneutral conditions (29°–30°C) (Reitman, 2018).

## Materials and Methods

### Study Subjects

Obese male mice (DIO-C57BL/6NTac, Cat DIO-B6-M, Taconic Biosciences, Germantown, NY) and lean male mice (C57BL/6NTac, Cat B6-DIOCONTROL-M, Taconic Biosciences, Germantown, NY) were received at 12 weeks of age. Animals were singly housed and given *ad libitum* access to a high-fat diet (HFD; D12492, 60 kcal% from fat; Research Diets, New Brunswick, NJ) or a low-fat diet (LFD; 10 kcal% from fat, Study 1- Cat #7012, Teklad, Madison, WI; Study 2- Cat# D12450J; Research Diets, New Brunswick, NJ) and water, unless otherwise noted. The DIO-C57BL/6NTac mice were fed HFD for 6 weeks before shipment. For Study 1, mice were maintained at thermoneutrality (29.1 ± 0.1°C) in a humidity (32 ± 2.4%)-controlled room on a 12-h light/dark cycle (11pm–11a.m.) at Cyclerion’s AAALAC accredited animal research facility. Study 2 was conducted at Biomeostasis’ facilities (La Penne-sur-Huveaune, France) where mice were maintained at thermoneutrality (30.0 ± 2°C) in a humidity (40–50%)-controlled room on a 12-h light/dark cycle (9a.m–9p.m). According to institutional guidelines, animals in both studies were housed in individually ventilated cages and provided enrichment including shepherd shacks, nestlets, and wooden bricks.

### Study Design

#### Study 1

##### Acclimation and Animal Selection

**FIGURE 1:**
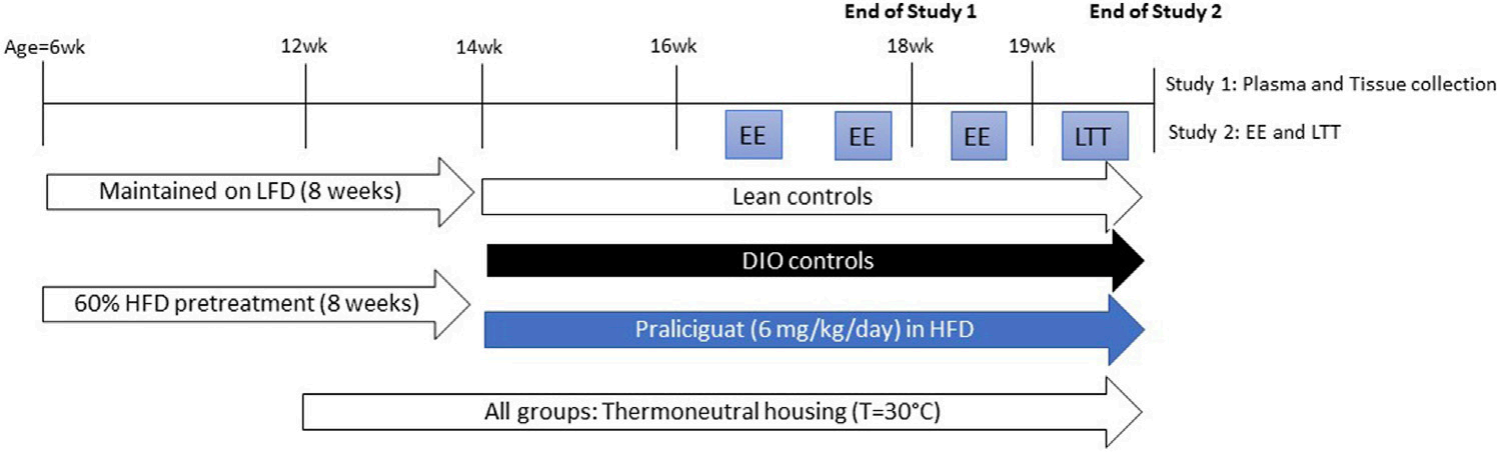
Experimental scheme for Study 1 and 2. DIO= Dietary-induced obese; EE=energy expenditure; HFD=high-fat diet; LFD=low-fat diet; LTT=lipid tolerance test.

Before study initiation, individually housed mice were acclimated to thermoneutrality for 2 weeks. During this period, body weight (BW) and food intake were determined twice weekly. After the 2 weeks, DIO mice were randomized and counterbalanced into 2 groups based on BW. A control group of same-age, lean mice, also housed at thermoneutrality, were maintained on LFD throughout the study (Figure 1).

##### Food Intake, Body Weight, and Body Composition Measurements

At treatment initiation, mice were either maintained on LFD (Lean control group; *n* = 8), HFD (DIO control group; *n* = 12), or were switched to HFD containing 6-mg/kg/day praliciguat (PRL DIO group; *n* = 12). Food was changed twice weekly and food intake was determined starting on day 6. Body weight was measured twice weekly. Whole-body composition was measured at baseline and weekly using quantitative magnetic resonance in awake animals (EchoMRI™, Houston, TX).

##### Praliciguat Exposure Sampling

On day 26, Mitra® microsamplers (Cat #10006, Neoteryx, Torrance, CA, United States) were used to collect blood samples from 6 praliciguat-treated mice between 10 and 11 a.m. (the last hour of the light cycle) to approximate C_min_ (minimum observed plasma concentration) and from the remaining 6 praliciguat-treated mice between 3 and 4 p.m. (4–5 h after the start of the dark cycle) to approximate C_max_.

##### Blood and Tissue Collection

On day 28, overnight-fasted mice received an oral dose of either vehicle (0.5% methyl cellulose/0.5% Tween 80) or praliciguat (6 mg/kg; to replace praliciguat lost during fasting). Two hours later, mice were sedated with isoflurane and blood was collected from the retro-orbital sinus into an EDTA blood collection tube (Cat #450474, Greiner Bio-One, Monroe, NC, United States) containing protease inhibitor cocktail (Cat #11 697,498 001, Roche Diagnostics, Indianapolis, IN, United States). Plasma was separated by centrifugation (3,000 × *g*, 20 min, 4°C). Following blood collection, animals remained sedated, were cervically dislocated, and tissue samples [liver, epidydimal white adipose tissue (eWAT) and skeletal muscle (SKM)] were collected, weighed, and flash frozen in liquid nitrogen, and then stored at −80°C until analysis. Mitra microsamplers were used to sample blood from the tail vein of awake, manually restrained praliciguat-treated mice for analysis of compound exposure.

##### Plasma Biomarker Assays

Colorimetric reagents were used to measure the following analytes: glucose (Cat #997-03001, Wako Scientific, Richmond, VA, United States), non-esterified fatty acids (NEFA) (Wako Scientific NEFA-HR, Richmond, VA, United States), triglycerides, and total cholesterol (Cat #T7532 and #C7510, Pointe Scientific, Canton, MI). The following analytes were determined by ELISA: IL-6 (Cat #K15069L-1, Meso Scale Discovery, Rockville, MD, United States), insulin, and C-peptide (Cat #90080 and #90050, Crystal Chem, Elk Grove Village, IL, United States). Homeostatic Model Assessment for Insulin Resistance (HOMA-IR) was calculated from fasting glucose and insulin values using the equation HOMA-IR = Fasting plasma glucose (mg/dl) × Fasting plasma insulin (ng/ml)/18.05 (Matthews et al., 1985).

##### Liver Neutral Lipid Determination

Ground liver (0.1 g) was homogenized (Omni Tip™ TH, Omni, Inc., Kennesaw, GA, United States) in 5% NP-40 substitute in water. Homogenates were incubated to extract lipids (80°C, 5 min) and centrifuged (3,000 × *g*, 20 min). Supernatants were analyzed for triglycerides (Pointe Scientific, Canton, MI, United States) and total protein (BCA Protein Assay Cat #23225, Thermo Scientific/Pierce Biotechnology, Rockford, IL, United States). Liver triglyceride content was expressed as mg triglycerides/g protein.

##### Gene Expression Analysis

Liver, skeletal muscle (SKM), and epididymal white adipose tissue (eWAT) samples were homogenized and processed using a QuantiGene™ sample processing kit (Cat #QS013, Affymetrix, Santa Clara, CA, United States) in accordance with manufacturer’s instructions. Tissue gene expression was measured using a QuantiGene 2.0 Plex Assay kit (Cat #QP1013, Affymetrix) and a custom-designed multiplex gene panel (Cat #QGP-150-M17011502, Thermo Fisher, Waltham, MA, United States). Analytes were measured using Luminex MAGPIX^®^ (Bio-Rad, Hercules, CA, United States). The median fluorescence intensity (MFI) was calculated for each gene target and normalized to the geometric mean expression of 3 housekeeping genes (Ppib, Tfrc, and Polr2a) that were chosen to match the target transcript abundance.

##### Phosphoprotein Analysis

Tissue samples from liver, SKM, and eWAT were homogenized and lysed with Cisbio lysis buffers containing protease and phosphatase inhibitors (PPI, Cat #78440, Thermo Fisher, Waltham, MA) using Qiagen TissueLyser LT with a 15-mm steel bead. Cisbio lysis buffer No. 2 (Cat #63ADK000ULB2, Cisbio, Bedford, MA) was used for phosphorylated vasodilator-stimulated phosphoprotein (pVASP) assay and Cisbio lysis buffer No. 1 (Cat #64KL1FDF) was used for phosphorylated ERK (pERK) and pAkT assays. After lysis, samples were kept on ice for 30 min and vortexed every 10 min. Samples were centrifuged and the supernatant protein concentration was determined (Pierce Cat #1861426, Thermo Scientific/Pierce Biotechnology, Rockford, IL, United States). Samples were then analyzed for phosphorylation of VASP, ERK, and AKT using Cisbio assay kits: pVASP (Ser239), Cat #63ADK065PEG; total-VASP, Cat #63ADK067PEH; pAKT (Thr308), Cat #64AKTPEG; pAKT (Ser473), Cat #64AKSPEG; total AKT, Cat #64NKTPEG; pERK1/2 (Thr202/204), Cat #64ERKPEG; and Total ERK1/2 Cat #64NRKPEG.

#### Study 2

##### Acclimation and Animal Selection

Before study initiation, individually housed mice were acclimated to thermoneutrality for 2 weeks. During this period, BW was measured 5 times and food was measured and changed daily. After the 2 weeks, DIO mice were randomized and counterbalanced into 2 groups based on BW, food intake, and fed blood glucose levels. A control group of same-age, lean mice, also housed at thermoneutrality, were maintained on LFD throughout the study (Figure 1).

##### Food Intake, Body Weight, and Body Composition Measurements

At treatment initiation, mice were either maintained on LFD (Lean control group; *n* = 12), HFD (DIO control group; *n* = 12), or were switched to HFD containing praliciguat (PRL DIO group; 6 mg/kg/day, *n* = 12). Throughout this study, food was weighed and changed daily. Body weight was measured twice weekly, except during the energy expenditure (EE) sessions where BW was measured daily. Whole-body composition was measured at baseline and after 3 and 5 weeks of treatment using quantitative magnetic resonance in awake animals (Minispec Analyzer LF50, Bruker, Germany).

##### Praliciguat Exposure Sampling

Blood samples from the tail vein of awake, manually-restrained mice were collected with Mitra microsamplers on day 5 and day 38 [1.5 h after administration of praliciguat and before the lipid tolerance test (LTT)].

##### Energy Expenditure Assessment

Twelve hours before each EE assessment, BW and body composition were determined, and mice were single housed in physiocages (Panlab/Harvard apparatus) for acclimation. Afterwards, metabolic parameters (oxygen consumption [VO_2_] and carbon dioxide production [VCO_2_]) were recorded at 1 measure/sec for 3 min every 30 min (mL/min/kg lean mass) for 48 h using METABOLISM V3.0 software (Panlab/Harvard apparatus), pausing at each 24-h interval to weigh the animals, replace food, and clean the physiocages. EE (kcal/day/kg lean mass) and fat oxidation (g/day/kg lean mass) were calculated based on the VO_2_ and VCO_2_ values.

##### Lipid Tolerance Test

Overnight-fasted mice received an oral dose of either vehicle or praliciguat (6 mg/kg) on day 38 to replace praliciguat lost during fasting. Ninety minutes later, blood was sampled to measure basal levels of triglycerides and NEFA). Thirty minutes later (2 h postdose), mice received an olive oil challenge by oral gavage (10 μL/g of BW) and additional blood (150 µL/sample) was collected at 1.5-, 3-, and 5-h post-challenge. The samples were added into centrifuge tubes pre-filled with 0.1 M EDTA (OmniPur^®^ EDTA, disodium salt, dihydrate, Sigma; 5 µL EDTA/100 µL blood). Blood was collected via the tail vein of awake mice, then plasma was separated by centrifugation (3,000 × *g*, 20 min, 4°C) and stored at –80°C until analysis of plasma triglycerides, as described above. Animals were euthanized at the end of the study with an overdose of anesthesia (Ketamine/Xylazine, 80/10 mg/kg) and organ collection.

### Statistical Analysis

Before statistical analysis, datasets were evaluated for outliers. All values > 2 standard deviations from the group mean were excluded. Data were analyzed using ANOVA. LTT data were analyzed using two-way repeated-measures ANOVA and EE data were analyzed using a mixed-effect analysis; both were followed by Dunnett’s test comparing against the DIO control group. All other data were analyzed using one-way ANOVA. Significant differences found by ANOVA tests were followed up by an appropriate post hoc test comparing against the DIO control group. A *p*-value of <0.05 was considered statistically significant. All data were analyzed and plotted using GraphPad Prism 9 software (San Diego, CA, United States).

## Results

### Effect of Praliciguat on Plasma Biomarkers

**FIGURE 2:**
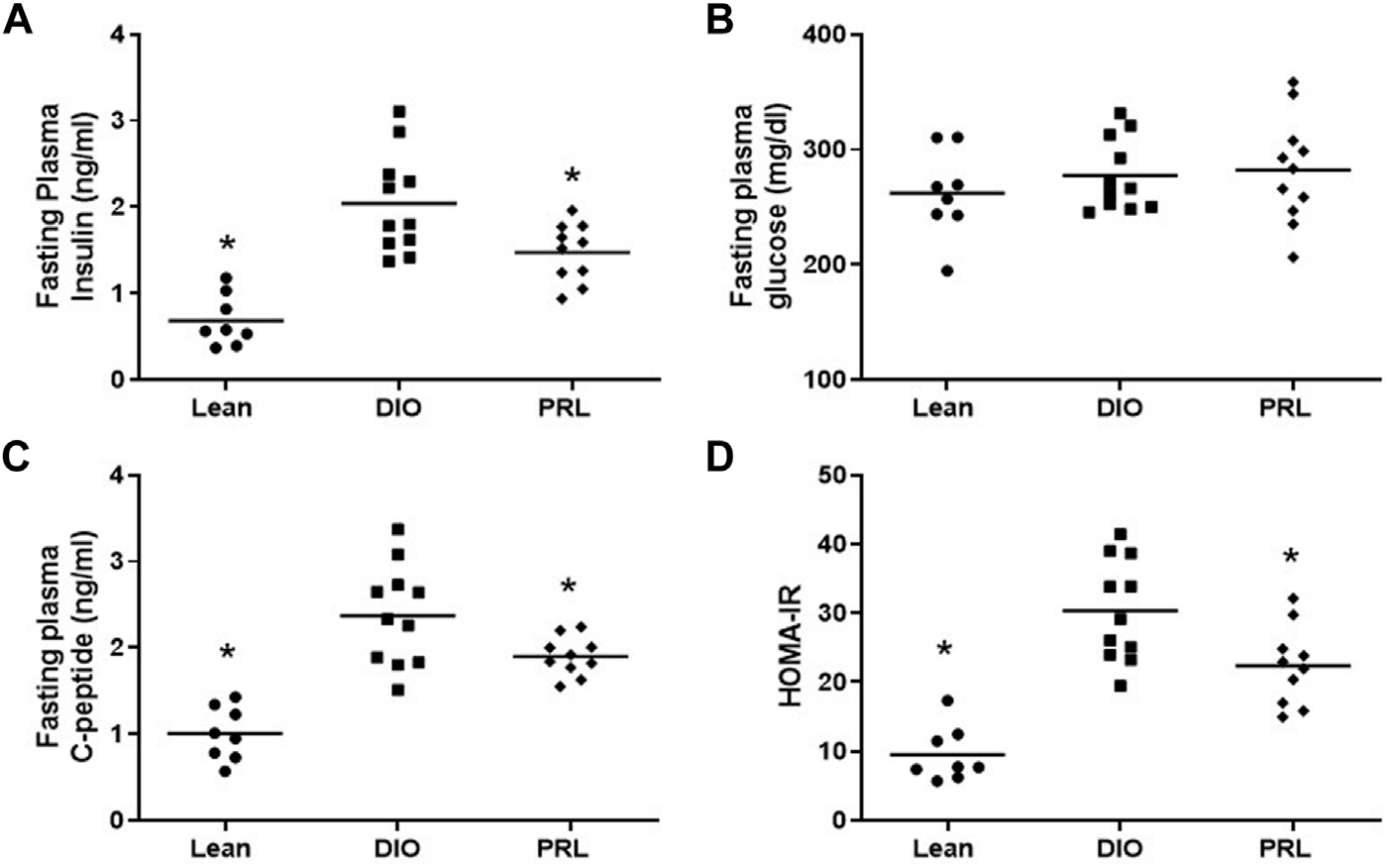
Effect of praliciguat (PRL) 6mg/kg/day on insulin sensitivity. Fasting insulin **(A)**, C-peptide **(C)**, and HOMA-IR **(D)**, but not glucose **(B)**, are lower in PRL-treated vs. DIO control mice. Data were analyzed by 1-way ANOVA followed by Dunnett's test with comparisons of each treatment group against the DIO control group. A <0.05 p-value is considered statistically significant (*). Individual animal data are shown; group mean is denoted with a line.

**FIGURE 3:**
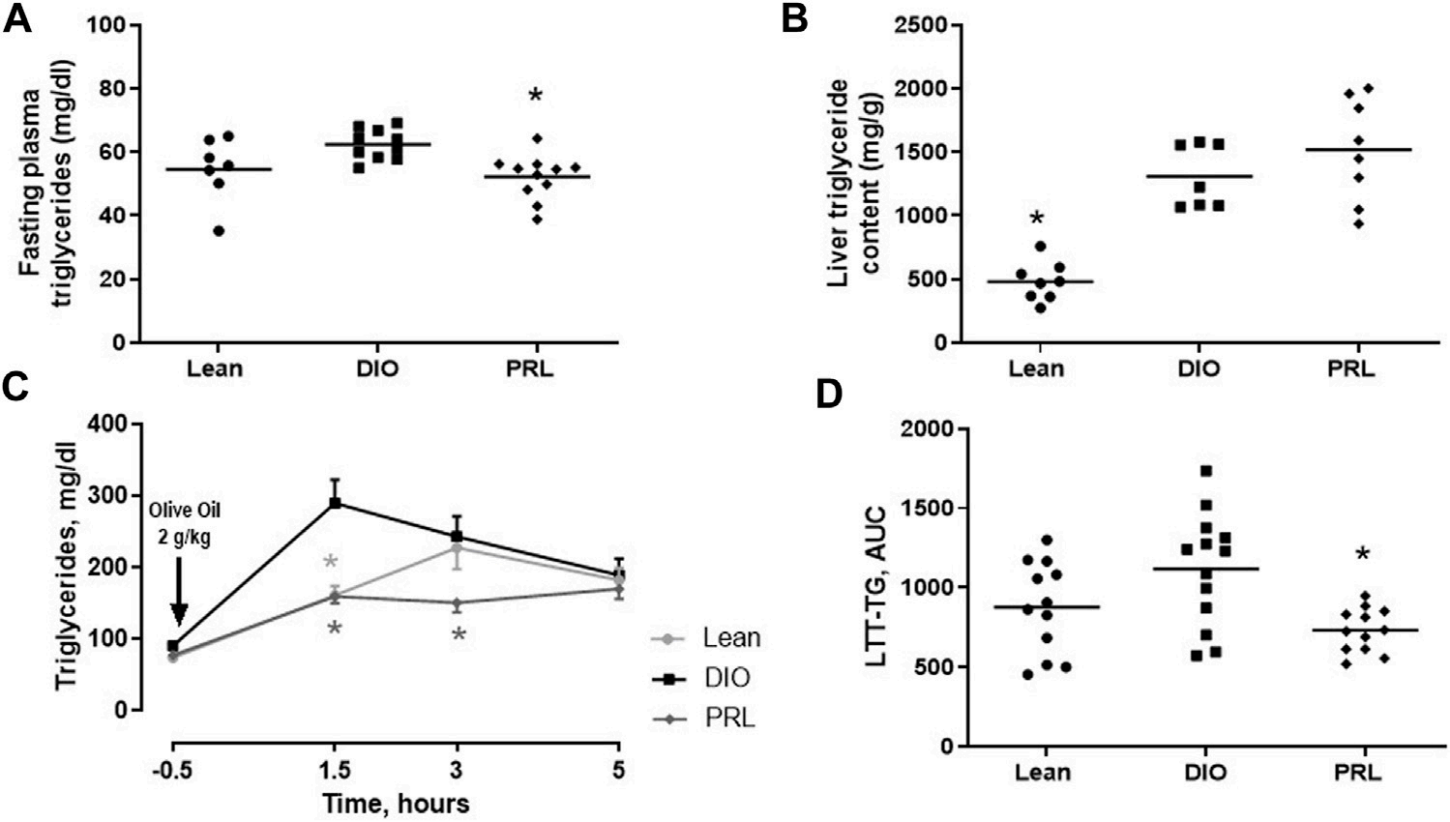
Effect of praliciguat (PRL) on triglycerides. In Study 1, PRL lowered fasting circulating triglycerides **(A)** but not liver triglyceride content **(B) **compared with DIO controls. In study 2, PRL lowered postprandial triglycerides represented as the time course of plasma triglycerides **(C) **or triglycerides area under the curve (AUC) **(D)**. Circulating triglycerides, liver triglyceride content, and AUC data were analyzed by 1-way ANOVA followed by Dunnett's test with comparisons of each treatment group against the DIO control group. Time-course data were analyzed by 2-way repeated measures ANOVA followed by Dunnett's test. At each timepoint, each treatment group was compared with the DIO control group. A <0.05 p-value is considered statistically significant (*). Data expressed as mean ±SEM or individual animal data are shown; group mean is denoted with a line.

In Study 1, circulating biomarkers of glucose tolerance and insulin sensitivity were measured after 4 weeks of treatment. Mean fasting plasma insulin was lower in lean (-67%) and praliciguat-treated (-28%) mice than in DIO control mice, while fasting plasma glucose was similar in all 3 groups (Figures 2A,B). Mean C-peptide was lower in lean (-58%) and praliciguat-treated (-20%) mice than in DIO control mice (Figure 2C). HOMA-IR was lower in lean (-69%) and praliciguat-treated (-26%) mice than in DIO control mice (Figure 2D). Fasting plasma triglycerides were lower in praliciguat-treated mice (-16%) than in DIO control mice (Figure 3A). Liver triglyceride content was lower in lean mice than in DIO control mice (-63%) and was similar in praliciguat-treated vs DIO control mice (Figure 3B). Throughout the experiment, there was no change in body weight, body composition, or food intake in praliciguat-treated mice compared with DIO control mice (Supplementary Figure S1).

In Study 2, plasma triglycerides and NEFA were measured in the LTT. In DIO mice, plasma triglycerides increased to an apparent maximum within 1.5 h post challenge (Figure 3C). After this timepoint, plasma triglycerides continually decreased but did not return to baseline by 5 h, the last timepoint measured. In praliciguat-treated DIO mice, plasma triglycerides were 45% lower 1.5 h post-challenge and 38% lower 3 h post-challenge than in DIO control mice. Triglycerides in lean mice continually increased for 3 h post-challenge and then decreased but did not return to baseline. In lean mice, plasma triglycerides were 44% lower at 1.5-h post-challenge than in DIO controls. LTT triglyceride area under the curve was similar in lean mice and was lower in praliciguat-treated mice (-63%) than in DIO control mice (Figure 3D). Additionally, fasting plasma triglycerides from the LTT were found to be lower in lean mice (-19%) and in praliciguat-treated mice (-13%) than in DIO control mice (Supplementary Figure S2A). Finally, the area under the curve of NEFA in the LTT was similar in all 3 groups (Supplementary Figures S2B). As observed in Study 1, there was no difference in body weight, body composition, or food intake between the praliciguat-treated mice and the DIO control mice (Supplementary Figure S3).

### Effect of Praliciguat on Gene Expression

**FIGURE 4:**
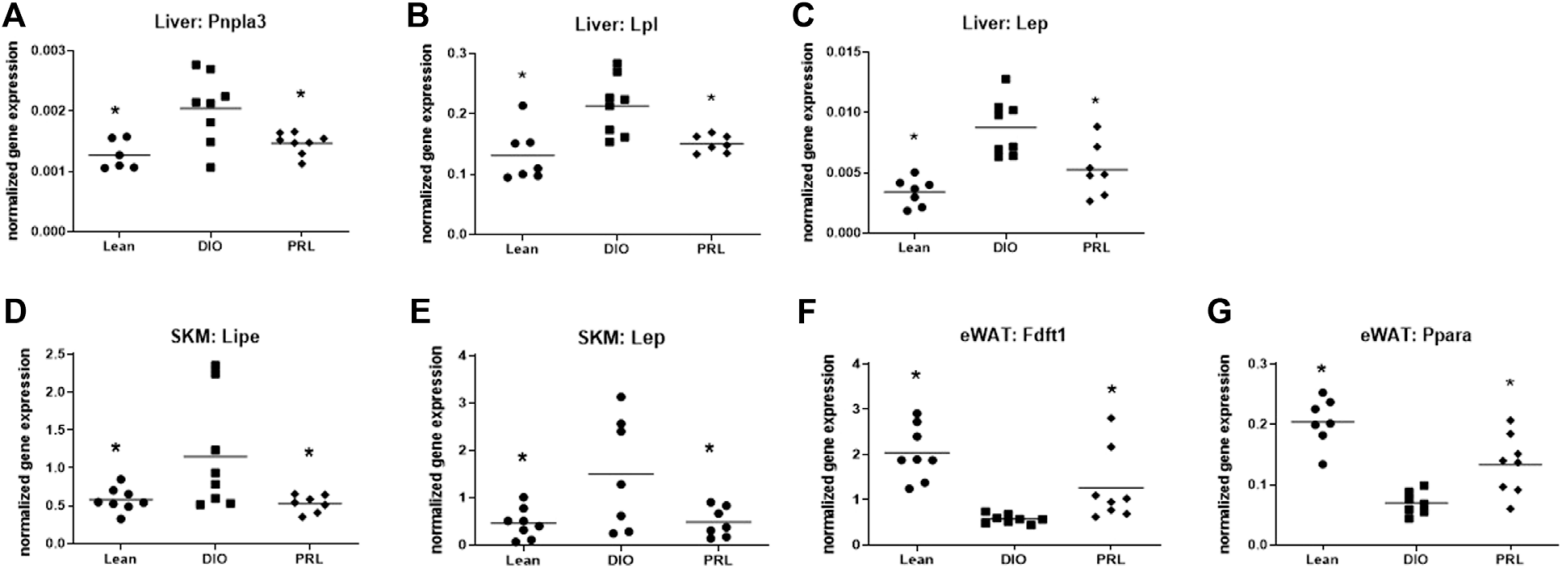
Effect of praliciguat (PRL) on lipid metabolism, energy metabolism, and fatty acid transport genes were analyzed in liver, skeletal muscle (SKM), and epidydimal white fat (eWAT) for gene expression using multiplex QuantiGene technology. Expression of liver Pnpla3 **(A)**, Lpl **(B)**, Lep **(C)**; SKM Lipe **(D)**, and Lep **(E)** were lower and expression of eWAT Fdft1 **(F)** and Ppara **(G)** were higher in PRL- treated mice than in DIO control mice. Gene expression data were normalized to housekeeping genes Ppib, Tfrc, and Polr2a. Significance was determined by 1-way ANOVA followed by Dunnett's test with comparisons of each treatment group against the DIO control group. A <0.05 p-value is considered statistically significant (*). Individual animal data are shown; group mean is denoted with a line.

Expression levels of several genes involved in lipid metabolism, energy metabolism, and fatty acid transport were altered by praliciguat treatment and were similar to those in the lean mice (Figure 4). Hepatic expression of *Pnpla3*, *Lpl*, and *Lep* was lower in praliciguat-treated mice than in DIO controls (Figures 4 A–C). Expression of *Lipe* and *Lep* was lower in skeletal muscle of praliciguat-treated mice than in DIO controls (Figures 4 D,E). The expression of epidydimal white adipose tissue *Fdft1* and *Ppara* was higher in epidydimal white adipose tissue of praliciguat-treated mice than in DIO controls (Figures 4 F,G).

**FIGURE 5:**
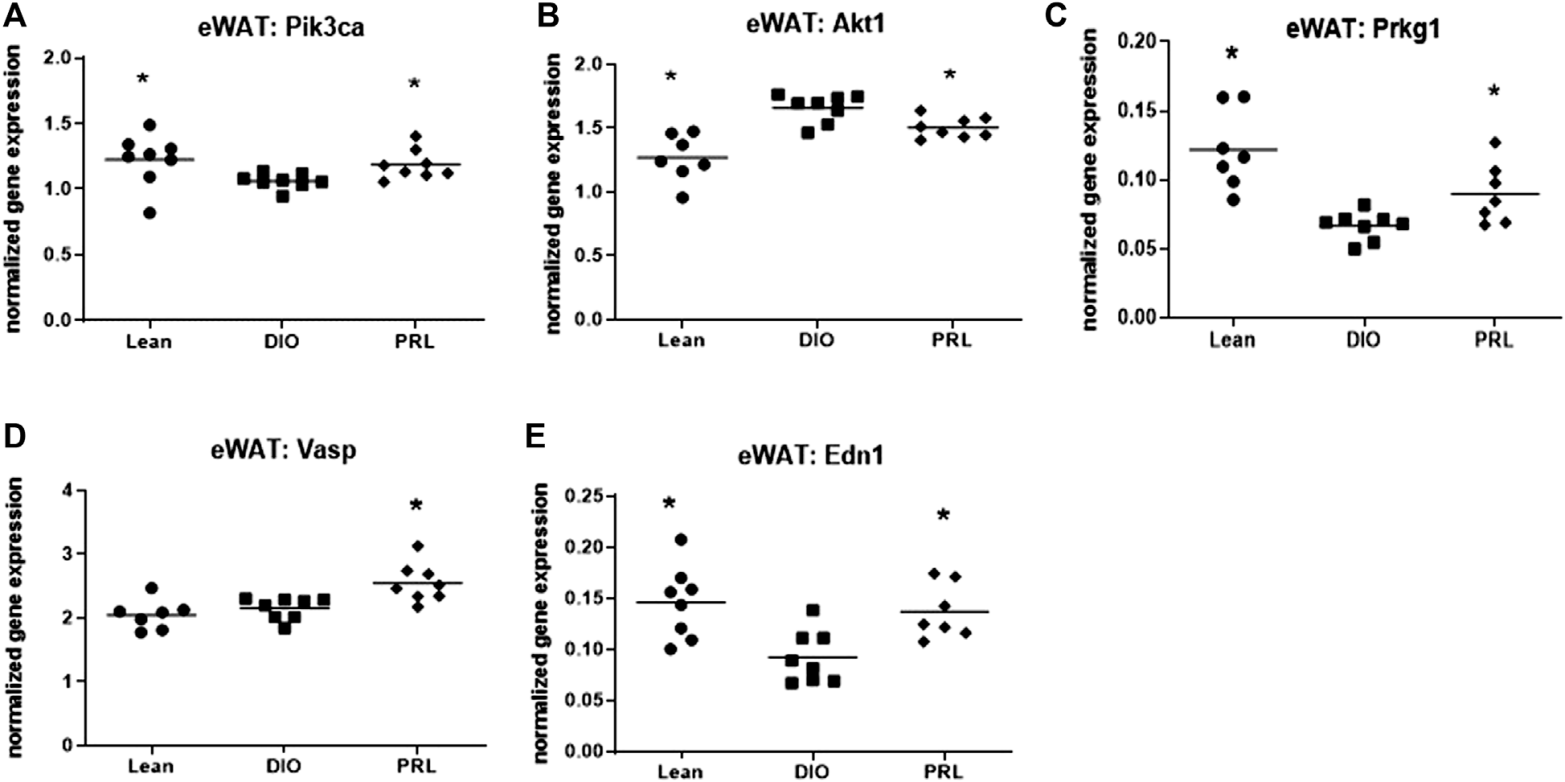
Praliciguat (PRL) modulates expression of genes in insulin and NO-sGC-cGMP signaling pathways. Epidydimal white adipose tissue (eWAT) samples were analyzed for gene expression using Multiplex QuantiGene technology for Pik3ca **(A)**, Akt1 **(B)** Pkrg1 **(C)**, Vasp **(D)**, and Edn1 **(E)**. Gene expression data were normalized to housekeeping genes Ppib, Tfrc, and Polr2a. Significance was determined by 1-way ANOVA followed by Dunnett's test with comparisons of each treatment group against the DIO control group. A <0.05 p-value is considered statistically significant (*). Individual animal data are shown; group mean is denoted with a line.

Several genes involved in insulin and NO-sGC-cGMP signaling were impacted by treating DIO mice with praliciguat and resulted in similar expression levels as lean mice (Figure 5). Expression of *Pik3ca*, *Prkg1*, *Vasp*, and *Edn1* were higher while *Akt1* was lower in eWAT of mice treated with praliciguat vs. DIO controls. The level of gene expression in praliciguat-treated mice was similar to lean mice with the exception of *Vasp*, which was higher compared with both DIO and lean control mice (Figure 5).

**FIGURE 6:**
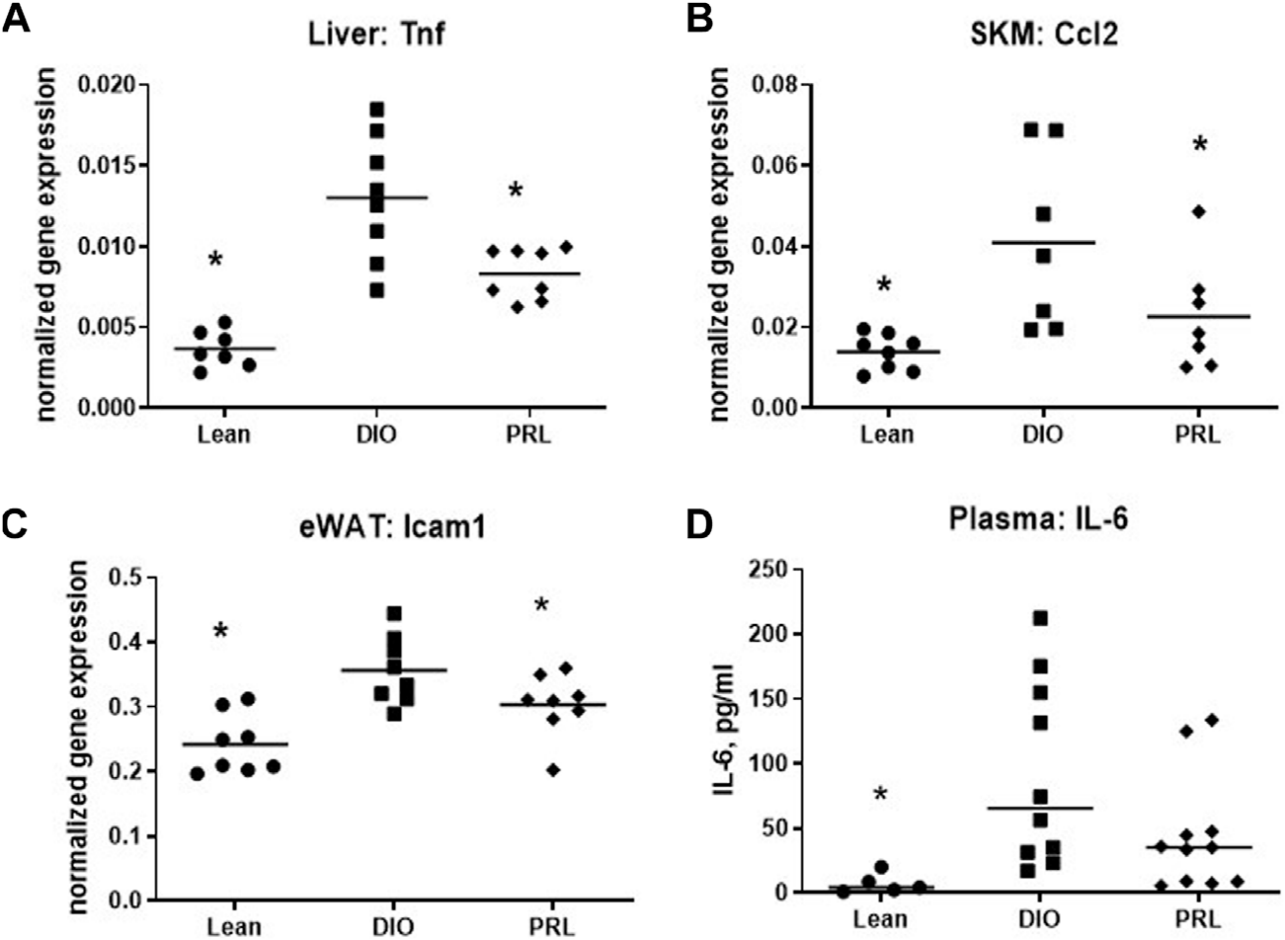
Praliciguat (PRL) modulates expression of inflammatory genes. Liver Tnf **(A)**, skeletal muscle Ccl2 **(B)**, and WAT Icam1 **(C)** were lower in PRL-treated mice than DIO controls. Gene expression was analyzed using Multiplex QuantiGene technology. Gene expression data were normalized to housekeeping genes Ppib, Tfrc, and Polr2a. Plasma samples **(D)** were analyzed for IL-6 by ELISA. Significance was determined by 1-way ANOVA followed by Dunnett's test with comparisons of each treatment group against the DIO control group. A <0.05 p-value is considered statistically significant (*). Individual animal data are shown; group mean is denoted with a line.

Expression of 3 genes involved in inflammation, including *Tnfα* in liver, *Ccl2* in skeletal muscle, and *Icam1* in eWAT was lower in praliciguat-treated mice than in DIO control mice (Figures 6A–C) and was also lower in lean mice. Plasma IL-6 was measured as an index of systemic inflammation. Compared with DIO controls, plasma IL-6 was lower in lean mice (-93%) and trended to be lower in praliciguat-treated mice than in DIO control mice, although this effect did not reach statistical significance (-60%, *p* = 0.06, Figure 6D).

### Impact of Praliciguat on Cell Signalling

**FIGURE 7:**
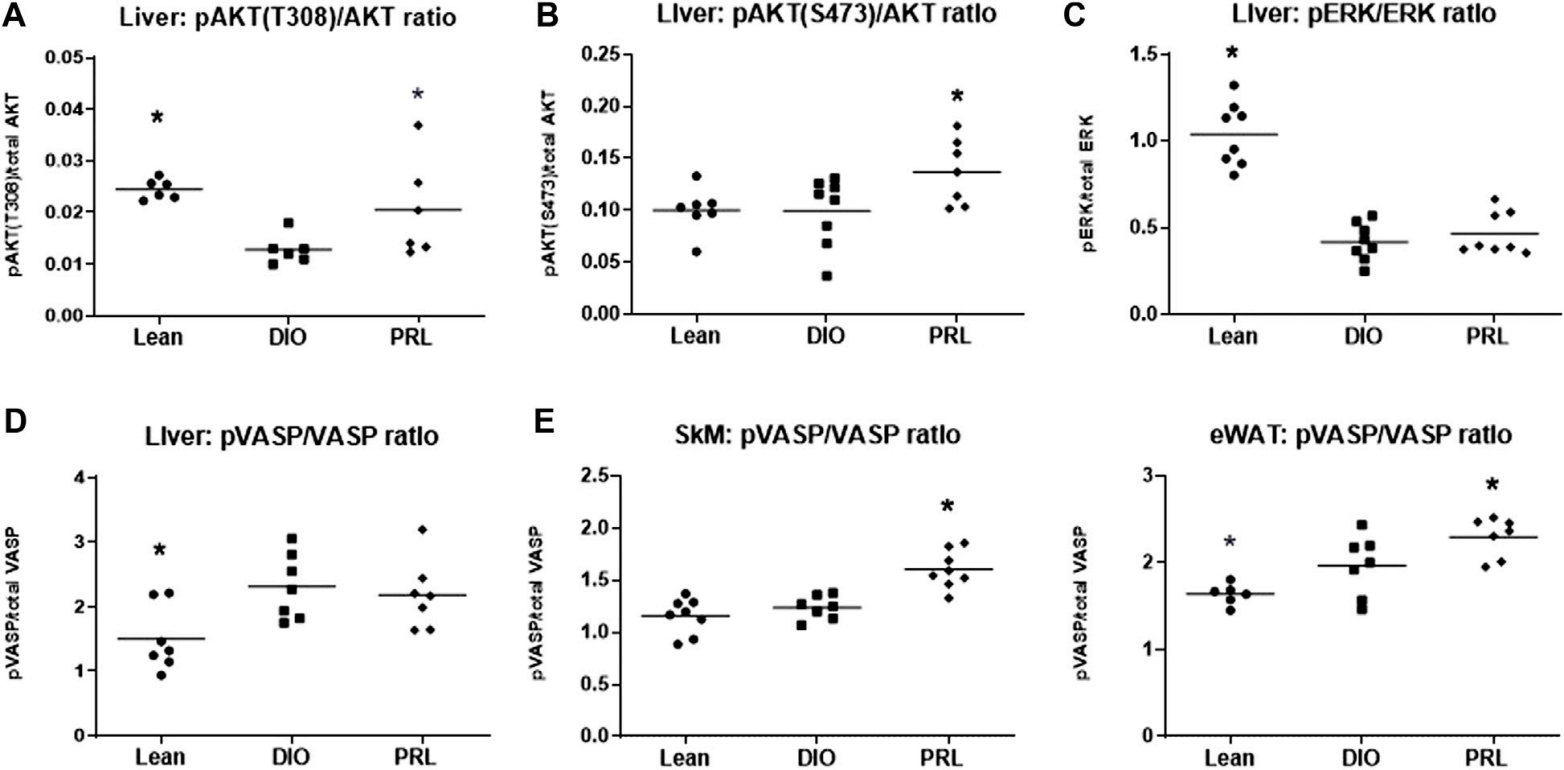
The effect of praliciguat (PRL) on phosphorylated proteins in the insulin signaling pathways in the liver were analyzed for **(A)** pAKT (T308), **(B)** pAKT (S473), **(C)** pERK and their respective total protein expression using HTRF assay. The effect of Praliciguat on pVASP/VASP was analyzed in insulin-sensitive tissues; **(D)** liver, **(E)** skeletal muscle (SKM), and **(F)** eWAT. Tissue samples were analyzed for pVASP and total VASP using HTRF assay. Statistically significant difference is based on 1-way ANOVA followed by Dunnett's test with comparisons of each treatment group against the DIO control group. A <0.05 p-value is considered statistically significant (*). Individual animal data are shown; group mean is denoted with a line.

The effect of praliciguat on insulin pathway signaling proteins was investigated using pAKT as a marker of the PI3K pathway activity and pERK as a marker of the MAPK pathway activity. Phospho-AKT(T308) and pERK signaling were lower in DIO controls than in lean mice (Figure 7). pAKT(T308) and pAKT(S473) but not pERK levels were higher in mice treated with praliciguat than in DIO control mice. The effect of praliciguat on pVASP, a key downstream signaling molecule of the sGC pathway, was also evaluated. In SKM and eWAT, pVASP levels were higher in DIO mice treated with praliciguat than in DIO control mice. This effect was not observed in the liver (Figure 7).

### Effect of Praliciguat on Energy Expenditure

**FIGURE 8:**
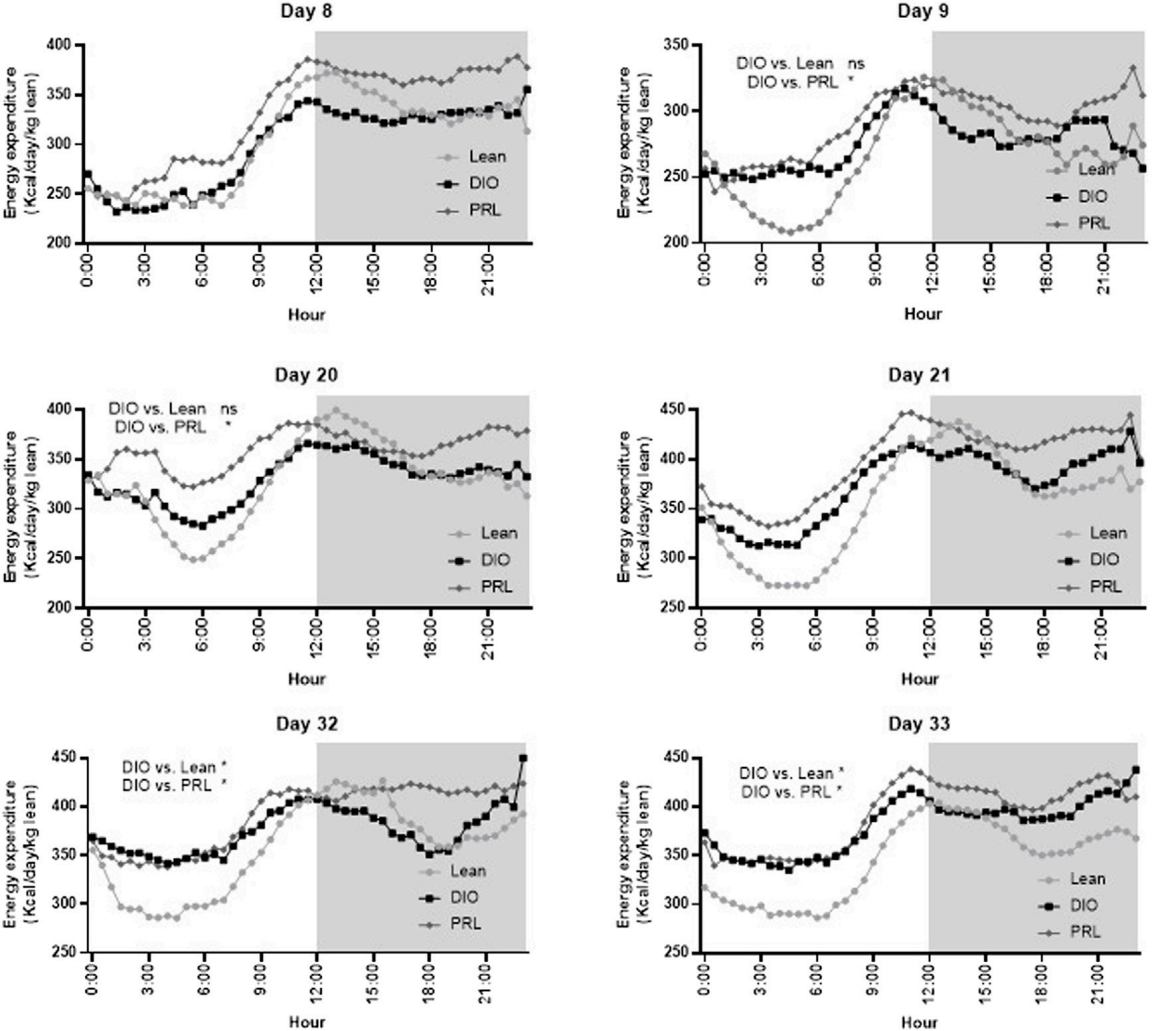
Praliciguat (PRL) increased energy expenditure in DIO control mice. Energy expenditure was analyzed by a mixed-effect analysis followed by Dunnett's test comparing against the DIO control group. On Day 8 and 21 there was no overall effect of group and thus a Dunnett's test was not conducted. The gray shaded area signifies the dark cycle. A <0.05 p-value is considered statistically significant (*). ns=not significant. Data are expressed as mean.

EE was assessed by indirect calorimetry measuring O_2_ consumption and CO_2_ production and was calculated as kcal/day/kg lean mass (Figure 8). On days 8 and 21, there was a significant effect of time but not group or interaction and thus no post-hoc test was performed. On days 9, 20, 32, and 33 there was a significant interaction or main effect of group and subsequent Dunnett’s tests were performed. On days 9 and 20, EE was higher in praliciguat-treated mice than in the DIO control mice and lean mice. During the final bout of indirect calorimetry (days 32 and 33), EE was higher in praliciguat-treated mice than in the DIO mice. Energy expenditure was lower in lean *vs*. DIO mice. Fat oxidation (g/day/kg lean mass), calculated from the indirect calorimetry data, was higher in praliciguat-treated mice on days 8, 9, 20, and 21 than in DIO control mice; on day 32 and 33 there was no difference between praliciguat-treated and DIO control mice. Fat oxidation was lower in lean mice than in DIO control mice across all timepoints (Supplementary Figure S4).

## Discussion

The objective of these studies was to explore the effect of praliciguat in a nonclinical model of metabolic syndrome that mimics some of the hallmarks of human metabolic syndrome, including increased adiposity, insulin resistance, and elevated triglycerides. Praliciguat, a clinical-stage sGC stimulator, has previously been shown to decrease blood pressure, reduce inflammation, and protect against end-organ damage in nonclinical disease models relevant to metabolic syndrome (Tobin et al., 2018; Shea et al., 2020). We studied a mouse DIO model characterized by increased adiposity and concurrent insulin resistance. We fed DIO mice HFD that was formulated with or without praliciguat. In both studies, the resulting blood exposure achieved with praliciguat-formulated HFD (0.4–0.9 nM; data not shown) was within the range of the concentrations measured in a clinical study in humans with type 2 diabetes and hypertension (Hanrahan et al., 2018; Hanrahan et al., 2020b).

It has been reported that in DIO mice, increasing cGMP via sGC stimulation or phosphodiesterase (PDE5)-inhibition increases insulin sensitivity, thus alleviating some of the consequences of metabolic syndrome (Hoffmann et al., 2015); however, in these previous studies, because body weight gain was lower in compound-treated animals, the metabolic benefit could not be fully attributed to sGC stimulation or PDE inhibition. Lack of weight gain on a HFD may be due to lower food intake or to higher EE. The potential mechanism for higher EE is brown adipose tissue (BAT) activation. Engagement of the sGC and natriuretic peptide pathways, which converge on the cGMP-protein kinase G (PKG) pathway, have both been demonstrated to activate BAT (Kimura et al., 2021). In the current study—and in contrast to what has been reported at standard housing temperature conditions—when DIO mice are housed at thermoneutrality, activation of the cGMP-PKG pathway via praliciguat treatment does not elicit a change in body weight, body composition, or food intake compared with DIO control mice (Supplementary Figures S1, S3). Therefore, when mice are housed in an environment that more closely matches human physiology, the effects observed in the current set of studies are independent of any beneficial effect of lower body weight and less adiposity.

### Impact on Inflammation

The anti-inflammatory effects of praliciguat have been well documented across multiple disease models, including attenuated renal expression of *Ccl2*, *Tnfα*, *Nfκb*, and *Icam1* in the Dahl salt-sensitive rat model (Tobin et al., 2018; Hall et al., 2019; Shea et al., 2020). In the current study, anti-inflammatory effects of praliciguat were observed in multiple insulin-sensitive tissues (liver, skeletal muscle, and eWAT). Additionally, plasma IL-6 levels, a clinically relevant biomarker of systemic inflammation (Ridker, 2016), trended lower in praliciguat-treated mice, suggesting lower systemic inflammation. Praliciguat’s broad anti-inflammatory effects across many tissues are likely due to its extensive tissue distribution (Tobin et al., 2018) and the previously documented anti-inflammatory effect of sGC stimulation on leukocytes (Ahluwalia et al., 2004; Tchernychev et al., 2021). These data add to the body of evidence that praliciguat has extensive anti-inflammatory activity.

### Impact on Insulin Sensitivity

In this study we demonstrated that animals treated with praliciguat had lower fasting insulin, C-peptide, and HOMA-IR but not fasting glucose in DIO mice, suggesting an improvement in insulin sensitivity. We propose that this improvement may be the result of increased cGMP production in the multiple tissues exposed to praliciguat (Tobin et al., 2018). Additionally, upregulation of *Pparα* in the eWAT has also been shown to improve insulin sensitivity (Verreth et al., 2004). To further investigate the observed improvements in insulin sensitivity, we explored several mediators of insulin signaling. VASP, a downstream mediator of the NO-cGMP signaling pathway, is involved in the restoration of insulin signaling (Kang et al., 2017). In our study, even though the impact of HFD on pVASP signaling was minimal, pVASP levels were higher in skeletal muscle and eWAT, but not in the liver of praliciguat-treated DIO mice vs. DIO control mice. Liver insulin signaling was assessed by determining pAKT (T308) and pAKT (S473) as markers of PI3K pathway activity, and pERK as a marker of MAPK pathway activity. Activation of both the T308 and S473 phosphorylation sites fully activates pAKT activity (Fayard et al., 2005) and DIO mice treated with praliciguat had higher pAKT (T308) and pAKT (S473) levels in the liver. We propose that praliciguat increased pVASP via increasing cGMP, leading to a reduction in HFD-induced inflammation and insulin resistance, leading to increased AKT activity (Kim et al., 2006). The MAPK insulin signaling pathway (pERK) was also explored and has been reported to play an important role in insulin signaling (Kim et al., 2006). In the liver of DIO control mice, we observed lower pERK signaling than in lean control mice, which was not restored by praliciguat treatment. Taken together, the data illustrate that the insulin signaling is perturbed in mice fed HFD, and praliciguat treatment improves some aspects of insulin signaling.

### Impact on Lipid Handling (Fasting/Basal and Postprandial Triglycerides)

Circulating, but not liver, triglyceride levels were lower in praliciguat-treated vs DIO control mice, suggesting improvements in lipid handling that occur outside or in concert with other insulin-sensitive tissues. It is likely that liver triglycerides did not change with praliciguat treatment because animals were maintained on HFD, and BW and food intake were the same as DIO controls. For instance, humans with fatty liver disease can successfully reduce liver fat, but only after diet modification and/or weight loss (Romero-Gomez et al., 2017). Therefore, the improvements observed in lipid handling in praliciguat-treated mice may be due to normalization of genes involved in lipid handling such as liver *Pnpla3* and *Lpl;* skeletal muscle *Lipe;* and epidydimal white adipose tissue *Pparα*. Therefore, we measured expression of metabolically relevant genes in the liver, skeletal muscle, and eWAT. In the liver, *Pnpla3* and *Lpl* were lower in praliciguat-treated mice vs. DIO controls. *Pnpla3* (patatin-like phospholipase domain-containing 3) codes for a protein that has been linked to the progression of fatty liver disease, including nonalcoholic steatohepatitis, and lies downstream of SREBP-1c, an important regulator of fatty acid synthesis (Bruschi et al., 2017; Basu Ray, 2019). Downregulation of hepatic *Pnpla3* has been shown to decrease liver fat content (Romeo and Savage, 2019). *Lpl* (lipoprotein lipase) codes for an enzyme involved in triglyceride release from circulating triglyceride-rich lipoproteins. In mice, liver-specific *Lpl* overexpression leads to a doubling of liver triglyceride content and insulin resistance (Kim et al., 2001), suggesting that a reduction in Lpl, such as observed with praliciguat treatment, might be involved in reducing insulin resistance or might be a marker of improved insulin sensitivity. In the skeletal muscle, Lipe (hormone sensitive lipase) was lower in praliciguat-treated mice than DIO control mice. *Lipe* codes for a lipase responsible for mobilizing stored fatty acids (Lan et al., 2019). Insulin and hormone-sensitive lipase are currently thought to be mutually regulated. In our study, *Lipe* gene expression was lower in SKM of praliciguat-treated vs DIO control mice, which may be linked to the lower fasting plasma insulin levels observed. Therefore, as mice become more insulin sensitive, less *Lipe* is needed to release fatty acids from tissue. Further, expression of *Pparα* (peroxisome proliferator-activated receptor alpha) in the eWAT was lower in DIO mice than in praliciguat-treated and lean mice. *Pparα* is known as the master regulator of lipoprotein and triglyceride metabolism genes (Han et al., 2017). In eWAT, upregulation of *Pparα* has been demonstrated to decrease hypertriglyceridemia (Verreth et al., 2004). Taken together, the improvement in lipid handling in DIO mice treated with praliciguat is likely due to the differential regulation of genes involved in lipid handling across multiple organs.

### Impact on Energy Expenditure

The majority of preclinical studies investigating compounds on metabolic health are performed at temperatures that are lower than the animal’s thermoneutral zone. Mice are typically housed in the 20°–23°C temperature range, which is several degrees lower than their thermoneutral temperature (29°–32°C). In standard housing conditions, mice will increase EE to stay warm. In the current study, under thermoneutral housing conditions, EE was higher in animals that received praliciguat than in DIO controls. Given that EE was normalized by the amount of lean mass, the changes in EE were not attributed to the abundance of muscle. Although EE was higher in praliciguat-treated mice, it did not translate into a difference in body weight, body composition, or food intake (Supplementary Figure S3). It has been demonstrated that an sGC stimulator can increase EE when animals are housed in standard housing conditions, which suggests that sGC stimulation can increase EE in both housing conditions (Hoffmann et al., 2015). The goal of this study was to mitigate the stimulation of BAT thermogenesis by housing mice in thermoneutrality; nevertheless, we cannot rule out that this increase in EE was due to a small activation of BAT or browning of eWAT. For the first 20 days of praliciguat treatment, fat oxidation was higher in praliciguat-treated mice than in DIO controls. However, this effect was no longer observed by the end of the study, suggesting that the animals had reached a new steady state of lipid handing and fuel utilization.

## Conclusion

In DIO mice housed at thermoneutrality, praliciguat demonstrated broad metabolic benefits such as increasing glucose utilization, improving triglyceride clearance in response to an oral lipid challenge, increasing insulin sensitivity, and lowering plasma triglycerides. The data highlight the potential of praliciguat to treat various diseases with underlying metabolic disease. Future experiments focusing on the effects of praliciguat in female DIO mice and aged DIO mice are important to further explore the metabolic effects of praliciguat.

## Data Availability

The original contributions presented in the study are included in the article/Supplementary Material, further inquiries can be directed to the corresponding author.
