# Supplementary figures and images for: Beneficial Metabolic Effects of Praliciguat, a Soluble Guanylate Cyclase Stimulator, in a Mouse Diet-Induced Obesity Model
Source: Front Pharmacol. 2022 Mar 4;13:852080. doi: 10.3389/fphar.2022.852080 (PMC8931041; doi:10.3389/fphar.2022.852080)

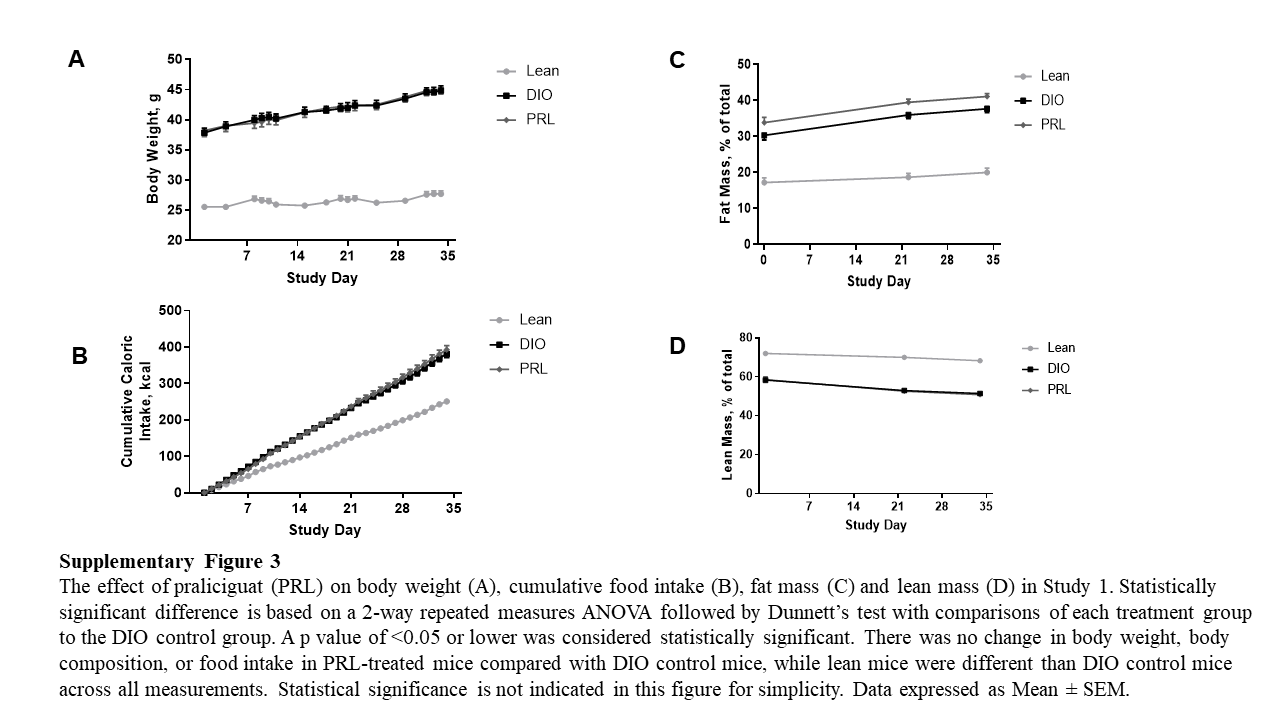

Supplement: Supplementary file 1 [file Image3.TIF]

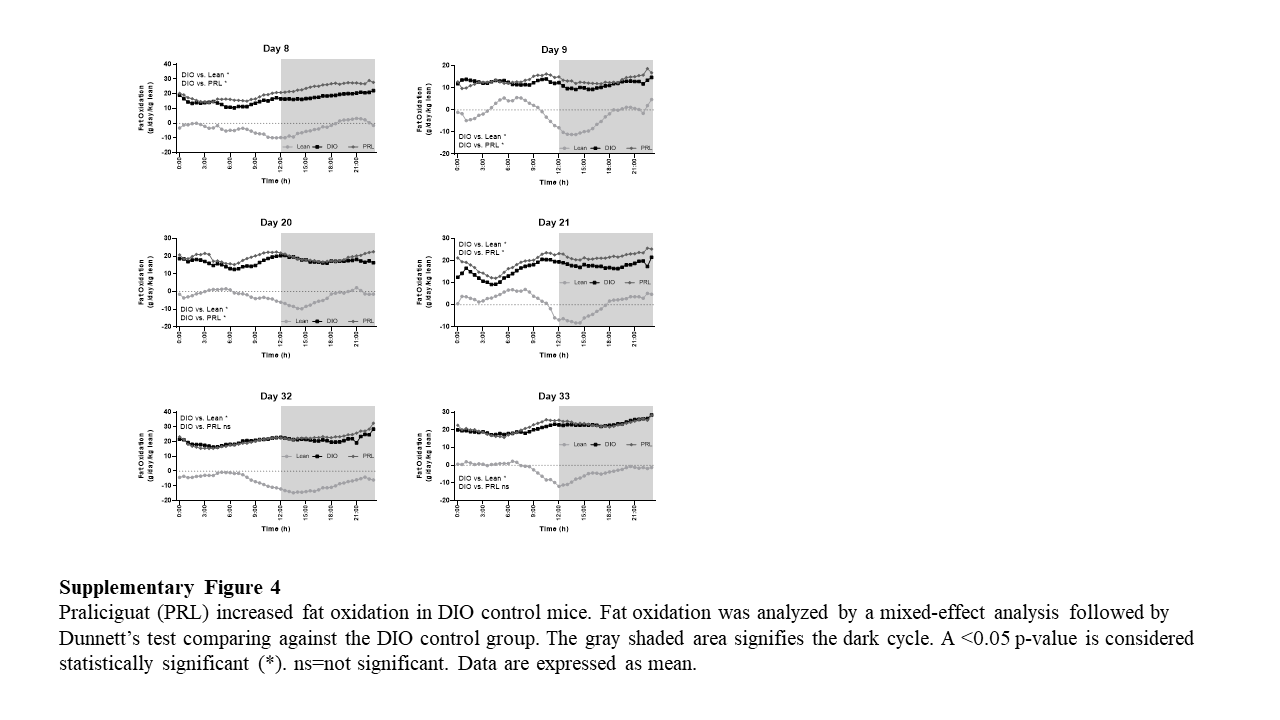

Supplement: Supplementary file 2 [file Image4.TIF]

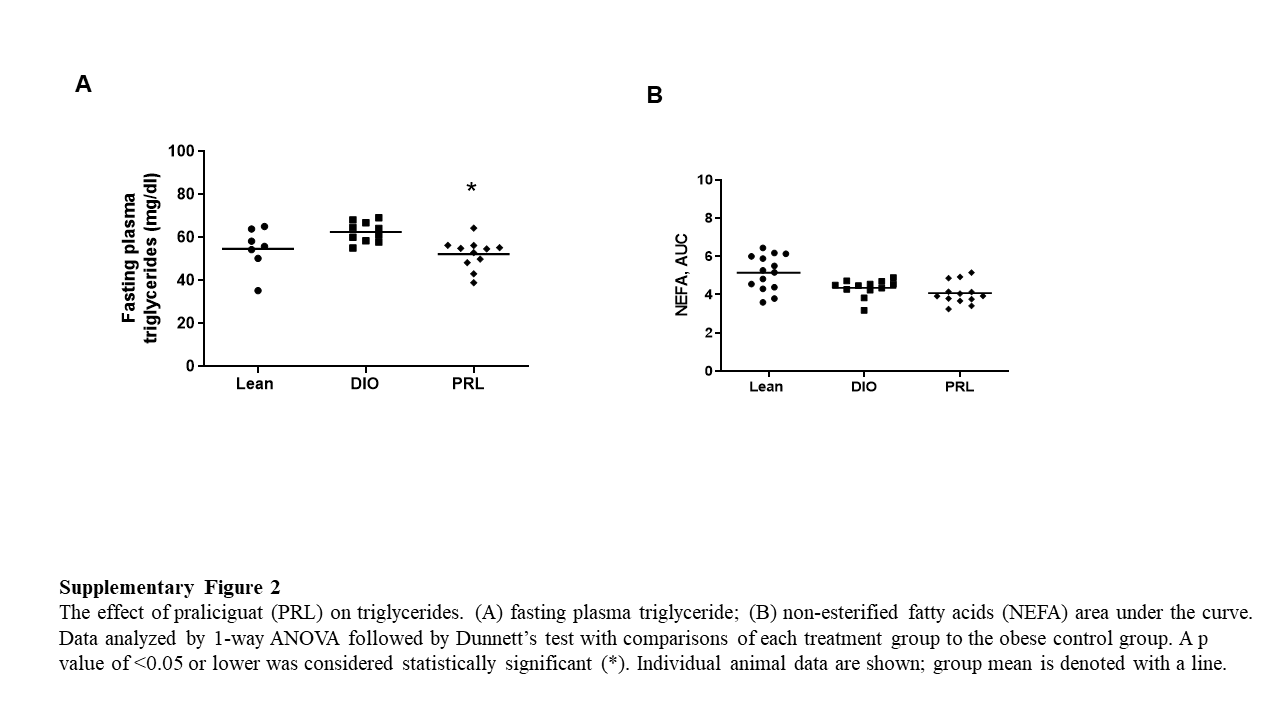

Supplement: Supplementary file 3 [file Image2.TIF]

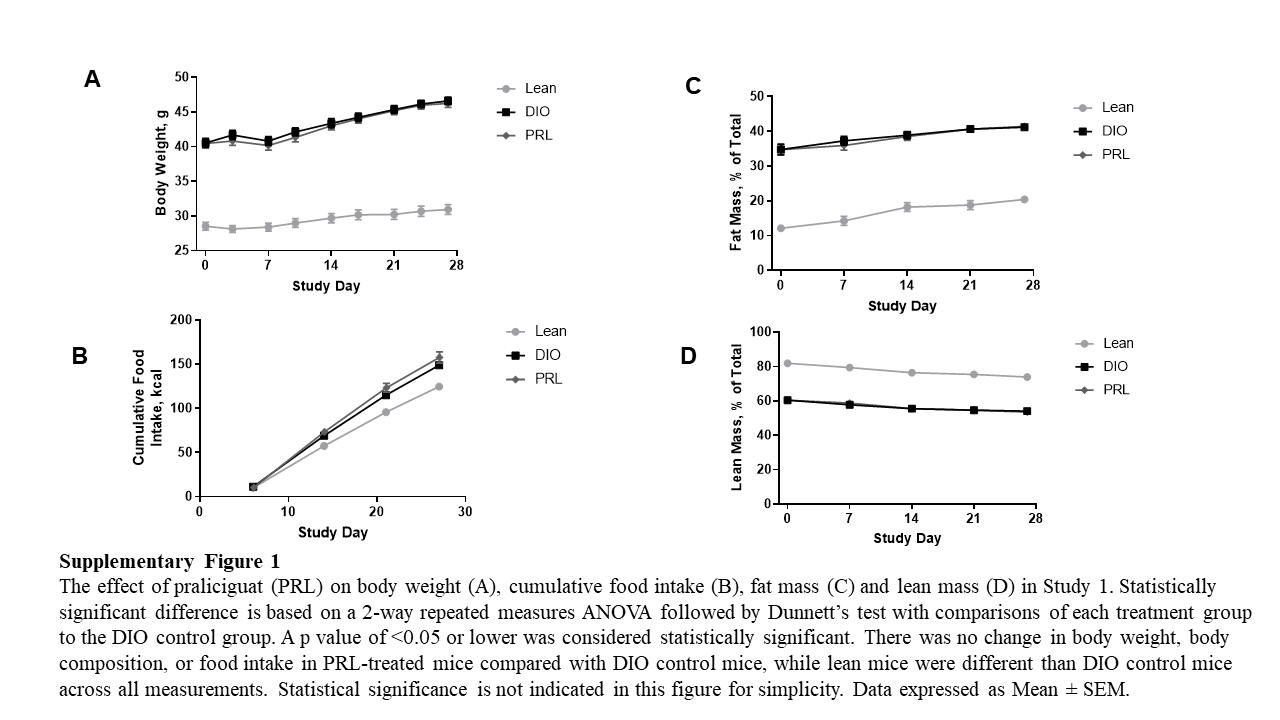

Supplement: Supplementary file 4 [file Image1.TIF]
